# Craftivism Between Nationalism and Activism in Ukraine and Belarus

**DOI:** 10.3389/fsoc.2021.659103

**Published:** 2022-03-02

**Authors:** Alla Myzelev

**Affiliations:** State University of New York, Geneseo, NY, United States

**Keywords:** craftivism, nationalism, Ukraine, Belarus, post-feminism, activism, activist art

## Abstract

This article outlines the history and significance of Craftivism in Eastern Europe. Using two case studies of artists it investigates the use of the craft language in Eastern Europe and its usability for activism. Do-It-Yourself culture, of which Craftivism is part, rejects the commercialism, gender norms and the conventional lifestyle in the Global North. Use of crafts as a language of political and social struggle allows to convey the message in a less confrontational but nevertheless very pertinent way. The craftivism is a successful language for the feminist political struggle in the Eastern Europe.

## Introduction

In the early days of the COVID-19 pandemic, a few friends (all Russian-speaking females in their 40s) decided to learn how to make masks. Connected through Zoom, we all found YouTube tutorial and bought or found attractive fabric. We expected to be done in one or two nights, after all, we all know how to sew. Making masks or making anything proved to be significantly more difficult than we expected. Two members of our small group decided to only provide support and not to try themselves after the first session. The rest of us persevered. About a week later, each of us had a mask. The masks that we made looked very “home-made”: the line of the thread was wavy, the shapes were not precise, and the ties needed adjustments. Moderately happy with the results, we were nevertheless puzzled by our lack of experience. During our last “debriefing” session, several of us wondered how come our handicraft skills were so abysmal. One of us noted that, in many ways, it was because of industrialization and even feminism. The second-wave feminists in the West tried to get away from housework and assume positions alongside men outside of the home. In the Soviet Union, while the ideology was different and women were encouraged to do handiwork and supplement what they could find in the stores with home knitting or sewing, most women had no time since they worked full time. Even if they knew how to sew, purchasing a sewing machine was difficult and constructing clothing was too time-consuming for most of them. Therefore revival of craftmaking in the 1990s and early 2000s required from Post-Soviet generation acquiring new skills.

This article deals with the use of DIY (Do-It-Yourself) culture in the political struggle of fourth-wave feminists in Ukraine and Belarus. For that purpose I use two case studies: Oksana Bruikhovetska’s series of suit jackets and Rufina Bazlova’ cross-stitch documentary series of Belarus protests. This article concentrates on the use of DIY culture in conjunction with the strategies of the third and fourth waves of feminism to understand how the aspects of textile art and craftivism assist in creating cultures of dissent. Both DIY culture and craftivism have direct and clear associations with needlework and craft. However, DIY culture, as will be explained further, acquired a much wider ramification throughout the 20th century to embrace alternative subcultures such as Situationist International or punk movement (Bennett and Guerra, 2018, 1–4). Craftivism, which appeared as a phenomenon significantly later, can be seen as one of the ways that DIY culture influences feminism, activism, and creativity. I argue that while DIY culture and craftivism in the Western discourse in the early 21st century often function on the border between post-feminism and fourth-wave feminism, within the discourse of Eastern European countries such as Ukraine or Belarus, craftivism should be understood as part of the radical feminist movement of the fourth wave.

## After the Third Wave: Post-Feminism and the Fourth Wave

Perhaps nothing exemplifies post-feminism better than the emotional tribulations of Bridget Jones in *Bridget Jones Diary* (2001). Created on the cusp of the millennium, it demonstrates that while feminism helped women to achieve equality, it left them wanting a fulfilling relationship. The feminist scholar Angela McRobbie argues that “post-feminism positively draws on and invokes feminism as that which can be taken into account, to suggest that equality is achieved, in order to install a whole repertoire of new meanings which emphasize that it is no longer needed, it is a spent force” (McRobbie, 2009, 13). Rosalind Gill agrees with McRobbie but adds that “... postfeminism is a sensibility is not fixed or relation on a singular understanding of the term; instead it ‘emphasizes the contradictory nature of postfeminist discourses and the entanglement of both feminist and anti-feminist theme within them’” (Gill, 2007, 149). I see the period of post-feminism that spans the late 1990s and early 2000s as a point when the media and overall prevalent opinion among women turned toward the ideas that feminism had achieved at least partial equality and, therefore, using femininity to one’s advantage was a matter of choice. Craftivists adapted the ideology of post-feminism and used elements of femininity to underscore the issue of post-feminist choice. I am arguing that craftivists in Eastern Europe are part of the fourth wave. The arrival of fourth-wave feminism roughly coincides with the increasing role of social media in spreading ideas. It predates the #MeToo movement, but the latter is definitely a part and in many ways a cataclysm of feminist revival. As Nicole Rivers notes, among all other waves, the fourth is complicated and multi-faceted. The main characteristics of this wave are the renewed pride in the term “feminism” along with extensive use of social and visual media in combination with in-person events. One of the main characteristics of contemporary fourth-wave feminism is its intersectionality and refusal to solely concentrate on issues that are traditionally considered feminine such as pay gap, child care, and media representations (Rivers, 2017, 134–35).

Finally, it is also important to note that while the metaphor of feminist waves is important for two metaphorical reasons: for underlying the presence of feminist movement from the 1840s to present and for practical discussions that allow for periodization, it is also fraught with contradictions. One of the main problems of the discussion of waves is the issue of what had happened in between upheaval. How the researchers think about ebbs of the feminist movement? Even more relevant to this discussion is the understanding that the feminist waves in the West are not universal and cannot be clearly mapped out to Eastern Europe, especially during the socialist regimes. One can even argue that while it is appropriate to talk about post-feminism and the fourth wave of feminism in Ukraine and Belarus, it is hard to argue that the second and third waves were comparable in the socialist countries (Zhurzhenko, 2001; Cerwonka, 2008; Zychowicz, 2011).

### DIY Culture

The return to craft-making in the West coincided with an interest in the DIY movement and the desire to take oneself out of the capitalist system of production and consumption to the extent that it was possible. Broadly speaking, DIY culture had existed as early as the development of the Industrial Revolution when people attempted to adopt the lifestyle that would take them away from the mainstream industrialized society (Frost, 2014, 1–15). George McKay links the new revival and redevelopment of DIY culture to the 1980s and 1990s when activists started organizing small protest actions that would fit within the non-conformist lifestyle (McKay, 1996). He defined it as “a youth-centred cluster of interests and practices around green radicalism, direct action politics, and new musical sounds and experiences… a kind of 1990s counterculture” (McKay, 1998, 2).

By the end of the 1990s, DIY culture became a prevalent type of expressing dissatisfaction with the culture of neoliberalism. The DIY spread into gardening, building, architecture, craft, music, and agriculture. It ranged from those who chose to make their clothing and bake their bread to combat capitalism through small deeds to those who learned to make furniture to undermine the monopoly of Ikea. One of the most prominent examples of the DIY that should be considered here is Riot Grrrl. As Hanna (Hanna, 1991) noted in the manifesto of the group, the main objection was again the fact that “concepts, ideas, and bodies also gain exchange value within a cultural context” (Riordan, 2001, 280). The resistance to the commodification of the female bodies, clothing, and music was at the core Riot Grrrl performances and DIY culture that they developed. Their music and the lyrics opposed commodification. Their performance venues created safe spaces for enjoy music and participate in celebration of femininity. All of this made the group unique part of the punk rock feminist history (Huber, 2010, 65–68). The DIY aspect of the group was expressed in the zines that the group published. The zines were home-made and home-printed magazines that expressed the esthetics and ideas of Riot Grrrl and were distributed to their fan base. The main aspect of the culture was to get people to do it themselves for themselves: “BECAUSE viewing our work as being connected to our girlfriends-politics-real lives is essential if we are gonna figure out how we are doing impacts, reflects, perpetuates or DISRUPTs the status quo (Hanna, 1991).”

In the early 21st century, the DIY movement had branched out into a subculture that was mainly insisting on negating capitalism and creating their lifestyle from scratch. A different brand of DIY feminism turned to the culture that emphasized femininity not as a way for men to objectify women but as a choice of being feminist. The third-wave feminism is concerned about the choice of agency and lifestyle. Many feminists of the third wave expressed themselves through feminine or girlie cultures as an attempt to compensate for the second waver’s refusal of femininity.

In the countries that comprised the Soviet Union, the feminist movement looked different. Feminism during the late Soviet period was expressed not as a desire for equality and ability to penetrate all aspects of the public sphere but in the need for the state to respect women’s differences. The demands that were expressed through several Samizdat publications mainly revolved around giving women longer maternity leaves, allowing women to be feminine, and recognizing that women usually work two shifts: work during the day and housework at night (Pető et al., 2002; Kaneva, 2017). Following Perestroika and subsequent rise of the independent countries such as Ukraine and Belarus, crafts industries had seen a rise in sales because the structure of women’s lives had changed. Women now seem to have choices of work although Marian Rubchak notes this seeming choice brought also further deterioration of the conditions of life and especially political disregard and inequality for women (Rubchak, 2009; Rubchak, 2012; Rubchak, 2019). Nevertheless, women had more access to making things themselves either to substitute for needed income or to practice what was of interest to them. During the 1990s and the early 2000s in Russia, Ukraine, and Belarus, women who were interested in needlecraft were not doing it as an expression of DIY but in a sense of opposing the capitalist system and the system supported by the governments. They were interested in crafts as hobbies and ways of self-expression.

### Craftivism

The term “craftivism,” a combination of two words: activism and craft, was coined by Betsy Greer (Greer, 2008). According to her 2003 definition, “craftivism is a way of looking at life where voicing opinions through creativity makes your voice stronger, your compassion deeper & your quest for justice more infinite” (Finn, 2009).^1^ Greer’s version of craftivism leaned toward liberal agenda and essentially geared toward using craft to express the concern for social issues that are faced by both men and women (Greer and Safyan, 2014, 2–4). One of the most famous examples of the craftivist actions was the use of pink pussy hats during the women’s march in 2016^2^. The craftivism project in the United States and Europe varied widely from small group projects that expressed the crafter’s political standpoint through large-scale craft projects such as creating a pink cosy for a public monument of a tank to demonstrate the anti-war view (Myzelev, 2009). Craftivism was also expressed through what became known as yarn graffiti. These are usually smaller size actions where objects made of needlecraft are exhibited as installations in public. They usually featured prominently on trees, houses, or public monuments. The use of the term “graffiti” suggests that sometimes the placement of needlework is not sanctioned by the institutions of power. Graffiti along with other craftivist actions have one tremendous advantage in comparison with other art projects. They are more relatable and usually accepted by the audiences and institutions as an expression of “soft” protest. For instance, the use of pussy hats with their pink colors and simple square design that gave wearers a look reminding of ponytail hairstyle prominent in young girls gave the march a more child-like, humorous, and at the same time memorable and attention-grabbing look. A similar strategy of craftivism was used as part of the protests of the Russian group Pussy Riot during their performance in Moscow’s Cathedral of Christ the Saviour in 2012. Their colorful, child-like clothing and unpolished musical performance express the culture of girlishness. This femininity and infantilization played into the perception of the West that was used for third-wave feminism. In Russia, it allowed not to take their message seriously and denigrate them to children (Myzelev, forthcoming).

Most of the studies of craftivism and political textile art are situated within Western discourse. There are only a handful of research projects so far that engage with textile art in Eastern or Central Europe in a political context and use feminist methodologies as an analytical lens (Buchczyk, 2014, 328–30; Asavei, 2019, 248; Kowalewska et al., 2018). This article looks at craftivism as a strategy of engaging, and resistance in Ukraine and Belarus to analyze how craftivism could potentially help in understanding the mechanism of political dissent but mainly to argue that seeming relatability and familiarity of the technique bares an unprecedented promise of opposition. Needlecraft’s associations of femininity and home-cultures seduce both the creators and the viewers. Fibers and textiles are among the most vulnerable materials that can deteriorate or be destroyed very easily, and therefore, they present no real danger in the physical sense. Thus, the combination of vulnerability and traditional association with felinity makes them much more relatable to the viewers and less threatening to the institutions of power. Used correctly, such ubiquitous objects as embroidered flowers or cross-stitched towels showing Belarus’s opposition to President Alexander Lukashenko became powerful tools of empathy, affect, and relatability.

Within the Western discourse, craftivism also came under scrutiny and criticism mainly for two reasons. Several scholars noted that craftivism’s practitioners are mainly women. The use of needlecraft by women for further political causes thus fits with the pre-existing stereotype of femininity lacking attributes of male protests such as violence, assertiveness, and critical oppositional stance (Groeneveld, 2010, 259; Pentney, 2008). The second line of critic comes from increasing commercialization of craft that especially gained speed since the 1990s in the West and also in Eastern Europe (as noted above). Craft theorists Bratich and Brush argue that, for crafts, to assume a significant position in and be practiced and recognized in academia is to be almost fully commodified (Bratich and Brush, 2011). Such an approach lies in the stress on the economic role of crafts and especially what the resurgence of craft means for larger Western business companies such as Hobby Lobby, Jo-Ann Stores, and others. However, one has to also take into consideration the human connection (Shiau, 2018) that craft provides for practitioners and customers. The gender and craft scholar Maria Elena Buszek along with the craft historian Glenn Adamson attempts in their respective publications to add theoretical grounding to the new revival of crafts as practice and especially to craftivism (Buszek, 2011, 5–8; Cronin and Robertson, 2011). They underscore craftivism’s ability to attract and connect people through its relatability and performativity (Oliver et al., 2010). Other responses to craftivism come from those historians who look at craftivism through postmodernism theory insisting on the importance of change from the modernist mode of production of a single artist in the studio to more socially aware practices of contemporary craftivists (Simpson, 2012, 248).

## Rufina Bazlova’s Cross-Stitch of Belarus Protests

Another example of the political charged DIY movement is the cross-stitch art created by the Belarus–Czech artist Rufina Bazlova. Her work uses traditional craft of Eastern Europe technique of cross-stitch embroidery to express and represent recent, 2020, Belarus protests. Using white background and red thread, Bazlova’s work repeats not only the traditional colors of embroidery but also the historical colors of the Belarus flag, black and white, which now is used as a symbol of anti-dictatorial protests.

**FIGURE 1:**
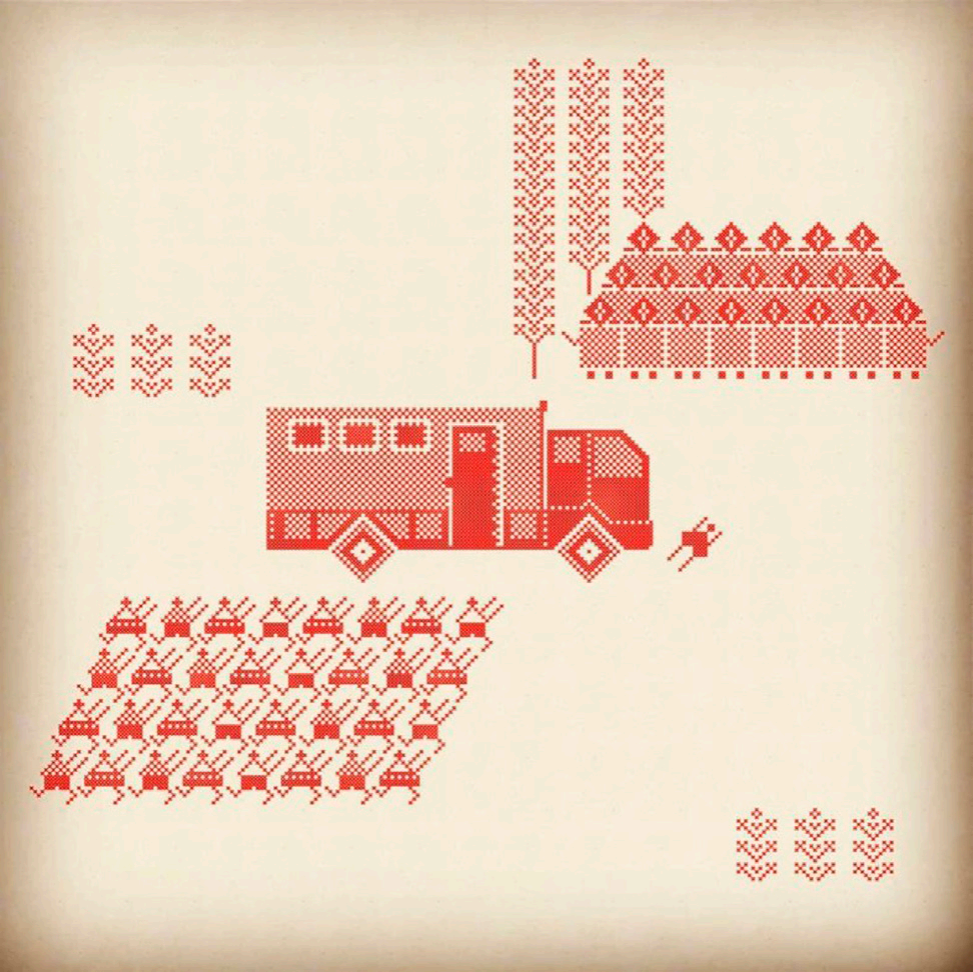
Rufina Buzlova, Avtozak hit a person during the demonstration, 2020, Cross-Stitch embroidery.

In the late summer and early fall of 2020, Belarus people rose to protest fifth re-election of their president Alexander Lukashenko. The latter came to power in 1994 and had remained the President of Belarus then. Out of five elections, only the first one is considered to be fair by the international authorities, while the rest of the elections, including the latest that took place on August 4–8, 2020, are considered falsified (Wesolowsky, 2020). From May 2020 to the time of writing of this paper, the protests in Belarus continued. Interestingly, women play a very important role in the protests, heading women’s marches in an attempt to minimize violence against the protestors. In response to the growing threat to Lukashenko’s regime, the latter asked the Russian President Putin for military and police help. Thus, many of Bazlova’s embroideries show the encounters between peaceful protestors and the members of Belarus (and/or Russian OMON). OMON is a Special Purpose Police Detachment trained to violently put down large national and local protests. In the embroideries, they are usually represented by the large faceless mass of marching or standing people. Bazlova’s language of embroidery is abstract enough to make the viewer stop and try to understand what and who is depicted and easily understood due to the depictions of the instances that were discussed and portrayed in the news. For instance (Figure 1), she described the images as follows.

These are not abstract topics—this is what I see or read in the news …. I simply sit and watch translation or read about what is happening in Belarus … and simply depict these stories. There are so many of them, so many thoughts in my head about what to do, but I simply do not have time to realize all of them. Yet, while I have an opportunity, I want to do it. *Avtozak* (A type of truck), which hit a person during the demonstration; or how some members of OMON side with people—this is not from my head, this is reality. (Antsiperova, 2020)

Thus, Bazlova transfers what she sees on social media or news into her narration using traditional feminine craft. Especially notable is the fact that while a lot of her work is embroidered, some are printed. Recognizing that handmade embroidery is a long and labor-intensive process, the artists use cross-stitch as a symbol of abstract representation to convey the meaning. Thus, the technique of embroidery becomes less important than the visual appeal and recognition of the white and red cross-stitch that becomes the recognizable element of her visual language. In this case, craftivism and political art become intertwined as two mutually supporting elements that help to further Bazlova’s political ideas. Interestingly, when asked, Bazlova notes that her art is not intended to move people to protest. Instead, she sees her art as an attempt to documenting and making sense of what is going in her homeland. Bazlova’ art is part of the fourth wave of feminism because of her embrace of the traditional domestic and feminine use of embroidery for political purposes. In addition, similar to many feminist artists of the fourth wave, she mainly spreads her message and her imagery on social media. The Internet then becomes the main vehicle for Bazlova’s art practice since she is physically removed from her homeland; she receives information online and disseminates her interpretation of that information also online. It is interesting, however, that she is still careful in her expression because of fear of retribution of Lukashenko’s dictatorship. In the interview, she noted “It is hard to say if I am encouraging people to protest—for that, I would be threatened by the article of the Law in Belarus—for now, I just react retrospectively” (Barkalova, 2020). Her reaction demonstrates that along with the positive ways that she uses online forms of communication, there are also issues such as online visibility that makes her think about physical security. The comment also betrays Bazlova’s understanding of her role in the process of Belarus protests. It betrays that her work of craftivism is conceived by her as “quiet activism” (Chuchvaha, 2020) as a reaction rather than a planned activist attack. In this case, there is a clear difference between an artist such as Bazlova and the feminist groups such as Femen or Pussy Riot whose protests are geared toward evoking strong responses.

### Oksana Briukhovetska's gender blended fashion

**FIGURE 2:**
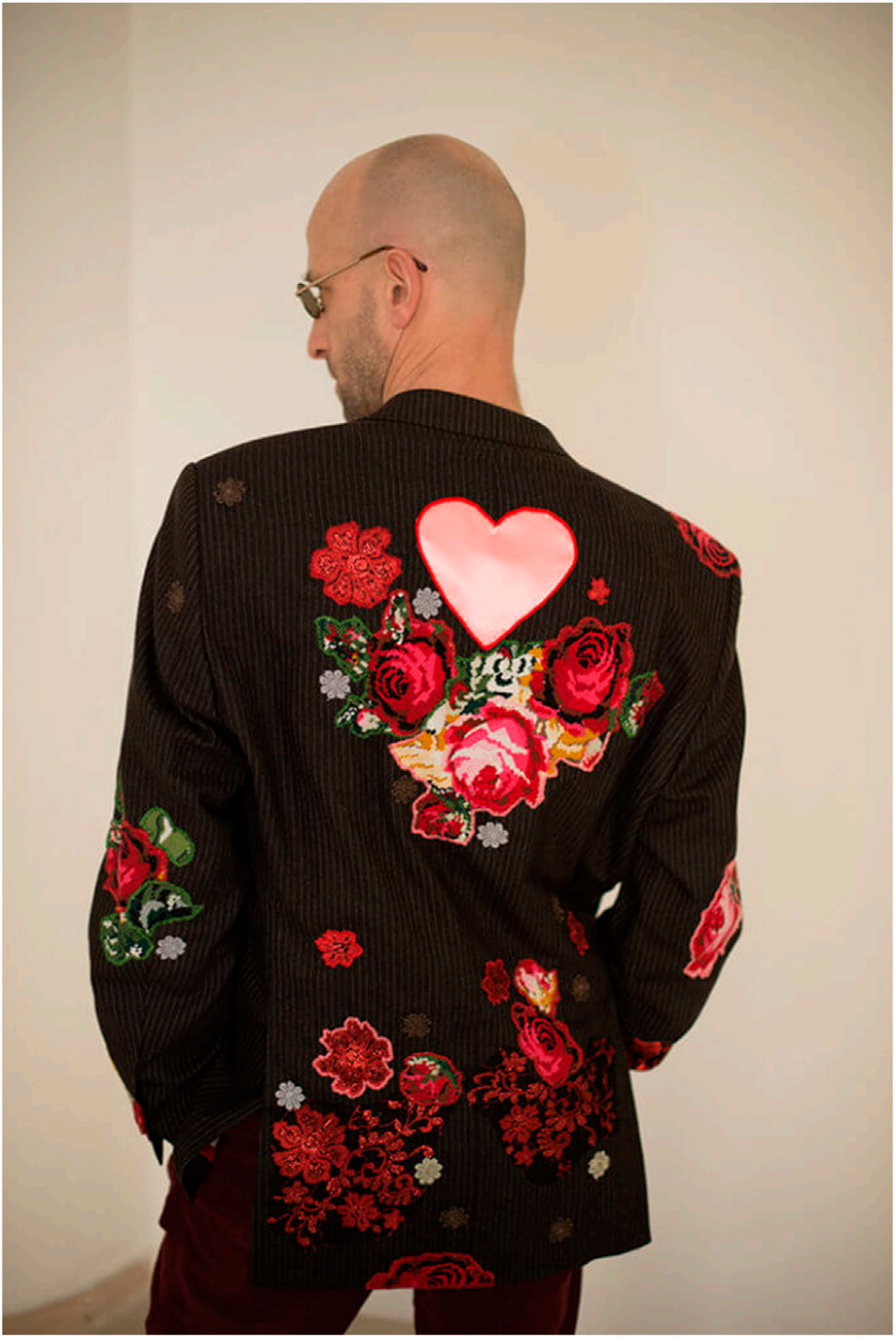
From Oksana Briukhovetska’s series, “Suit Jackets.”

For the 2017 exhibition “TEXTUS. Embroidery, Textile, Feminism,” the Ukrainian artist and curator Oksana Briukhovetska created a series of man’s jackets decorated with large appliqué pink, yellow, blue, and red flowers. She used traditional men’s black- and gray-striped jackets that are usually associated with the business world and male power (Figure 2). Then, cut-out and attached large pink and red flowers are usually associated with feminine fashion and femininity in general. Thus through the jackets, she created hybridity that includes both masculinity and femininity. Worn for the exhibition by both men and women, the jackets seem to be equally suitable for both male and female fashion and wear. Briukovetska, who also curated this exhibition in Kyiv, notes that she wanted to challenge the norms that govern the lives and men and women in Ukraine (Zlobina, 2017).

As with much other craftivist work that comes from Eastern Europe in the last five or so years, the ideas or ways of execution are not necessarily new. Many, including Briukovetska’s work, borrow from the language of feminist artists of the second wave, the 1970s, or craftivists of the 1990s and early 2000s. The *Suit Jacket* series, for instance, can be compared to both runway project and some gender-bending Western street fashion. Yet, the context of production and exhibition is very different. The fact that feminized men’s wear is still an artistic novelty and a craftivist project in Ukraine demonstrates the importance of challenging gender norms and stereotypes in the country, especially seeing the radical turn toward conservative nationalism in the Eastern European countries.

On the conceptual level, *Suit Jacket* is interesting in the sense that it both creates a unisex style that could be worn by both men and women and does not reflect the boyish look that a lot of unisex clothing tends to have. In other words, instead, fashion does not make women boys or men by offering T-shirts or boyfriends’ jeans but feminizes masculine fashion. At the same time, it also still functions with the heteronormative binary definitions of male/female or black/colorful. These works demonstrate the challenge of the norms but still retain the social normalization of feminine and masculine gender.

## Conclusion

The case studies that I presented use the approach of the DIY movement and to an extent craftivism to express the various concepts that have to do with gender and democracy in their particular countries. By using intersectionality as a guiding principle of their practice, they protest using material culture and craft as recognizable ways to be understood. Rufina Bazlova’s embroideries borrow from traditional national language of embroidery and remind the audiences about the traditionally feminine craft. Mainly mediated through the screens and deprived of the physicality of embroidery, her work becomes simplified, abstracted illustrations of the protests of Belarus. Her craft legacy makes her works much more effective than snapshots or naturalistic representations because of two factors. One, the embroidery pattern appeals to viewers and evokes the affective reactions. Second, abstracted cross-stitch prevents image saturation or desensitization that is often experienced by viewers. Bruikhovetska’s *Suit Jacket* series challenge gender norms through the language that is most familiar to both men and women—language of clothing and fashion. Using DIY esthetics, she works with ready-made objects such as jackets. Yet, because of her manipulation, the jackets acquire different, feminized meaning and attract attention of the gendered Ukrainian society.

This article demonstrates the beginning of the influences of fourth-wave feminism on art and protest in Eastern Europe. At the same time, it also shows that feminism, political protest, and craftivism are essentially intertwined and inform each other. It seems that, for Eastern Europe, at least the intersectionality of the fourth wave becomes the most important aspect since the feminist fight revolves not only around gender norms but also more importantly around survival or liberal (not neo-liberal) ideas and democracy.

## Data Availability

The raw data supporting the conclusions of this article will be made available by the author, without undue reservation.

## Footnotes

1 It is important to note that Greer’s definition of feminism is somewhat simplified.

2 “Pussyhat Project: Design Interventions for Social Change.” n.d. https://www.pussyhatproject.com/our-story.
